# A Real-Time Respiration Monitoring and Classification System Using a Depth Camera and Radars

**DOI:** 10.3389/fphys.2022.799621

**Published:** 2022-03-09

**Authors:** Shan He, Zixiong Han, Cristóvão Iglesias, Varun Mehta, Miodrag Bolic

**Affiliations:** Health Devices Research Group, School of Electrical Engineering and Computer Science, Faculty of Engineering, University of Ottawa, Ottawa, ON, Canada

**Keywords:** respiration rate, respiration patterns, IR-UWB radars, Kinect, contactless respiration monitoring, remote sensing

## Abstract

Respiration rate (RR) and respiration patterns (RP) are considered early indicators of physiological conditions and cardiorespiratory diseases. In this study, we addressed the problem of contactless estimation of RR and classification of RP of one person or two persons in a confined space under realistic conditions. We used three impulse radio ultrawideband (IR-UWB) radars and a 3D depth camera (Kinect) to avoid any blind spot in the room and to ensure that at least one of the radars covers the monitored subjects. This article proposes a subject localization and radar selection algorithm using a Kinect camera to allow the measurement of the respiration of multiple people placed at random locations. Several different experiments were conducted to verify the algorithms proposed in this work. The mean absolute error (MAE) between the estimated RR and reference RR of one-subject and two-subjects RR estimation are 0.61±0.53 breaths/min and 0.68±0.24 breaths/min, respectively. A respiratory pattern classification algorithm combining feature-based random forest classifier and pattern discrimination algorithm was developed to classify different respiration patterns including eupnea, Cheyne-Stokes respiration, Kussmaul respiration and apnea. The overall classification accuracy of 90% was achieved on a test dataset. Finally, a real-time system showing RR and RP classification on a graphical user interface (GUI) was implemented for monitoring two subjects.

## 1. Introduction

Contactless monitoring has several advantages in comparison with wearable technologies including the fact that it does not interfere with the subject's normal behavior, does not require any activation of the system or daily maintenance such as cleaning or charging batteries and does not require contact with the body. However, contactless monitoring using radars also has several disadvantages in comparison with wearables including reduced accuracy of monitored parameters and its dependence on the orientation of subjects and their location in the room, monitoring only in confined areas, difficulties in identifying the person being measured, or assigning the measured signals to the same person in multiple-person scenarios and interference from other moving people might affect the measurements of stationary people. These are serious problems that limit the widespread use of contactless devices in general and/or limit them to monitoring a single person. In this study, we tried to address several of these issues. In this article, we propose a system for contactless monitoring of respiration rate (RR) and respiration patterns (RP) of two subjects using three radars and a 3D depth camera (Kinect). The system continuously and unobtrusively monitors people when they are in the range of sensors. The main applications of our system include monitoring respiration patterns of patients with congestive heart failure (Tobushi et al., 2019), detecting drug overdose (Bachmutsky et al., 2020), monitoring inmates (Graichen et al., 2012), monitoring elders in their living environments (home, retirement home) and assisting in monitoring COVID-19 (Islam et al., 2021). However, this work is a proof of concept and has not yet been used in these applications.

We use inexpensive off-the-shelf components to build the system. Multiple radars are used to cover the whole area of the room. In addition, detection of the subject by multiple radars allows us to select the best signal from the radars reducing the dependence on the orientation of the subjects, their location in the room, or movement of other people on the accuracy of RR estimation. We do not record videos or utilize RGB videos in any way for privacy reasons. From the depth camera, we only use the skeleton information in the form of subject's joints coordinates and IDs. In addition, inaccuracies in RR estimation are further reduced by detecting motion artifacts and recognizing/classifying the abnormal breathing patterns before RR estimation.

Respiration rate is the number of breaths a person takes per unit of time and it is usually determined in practice by counting the number of times the chest rises or falls per minute. RR is one of four primary vital signs that can be used to assess an individual's general physical health. The current practice is to use contact-based RR monitoring methods in clinical and occupational setting. These methods are based on recording respiratory airflow, sounds, air temperature, and chest wall movements (Massaroni et al., 2019b). The drawbacks of these approaches include sensitivity to motion artifacts and often uncomfortable to wear limit their use for long-term monitoring.

Recently, contactless RR monitoring has become an attractive research topic. In Massaroni et al. (2019a), an RGB camera was used to monitor the subject's breathing pattern and RR at a distance of 1.2 m and the mean error between the estimated RR and the reference measurement was −0.01 breaths/min. In Chan et al. (2020), an infrared thermal camera was employed for contactless RR estimation within 1 m (0.4–0.6 m). The IR-UWB radar which has high resolution and good penetration can recognize the subtle movement of human body parts through clothing (Lazaro et al., 2010; Hu and Jin, 2016) and therefore it is widely used for contactless RR monitoring as well. The important component of radar-based RR estimation is detection and localization of human subjects. In Goldfine et al. (2020), a multiple-radar system was developed for subject localization and RR estimation. However, the focus of the article is on localization and RR estimation of a single subject. In Koda et al. (2021), frequency modulated continuous wave (FMCW) radar with 3 × 4 receiving and transmitting element was used to locate and estimate multiple people and their RR.

Respiratory patterns related to breathing disorders are commonly analyzed based on the respiratory signal collected by contact-based devices, including respiratory inductance plethysmography (RIP) and polysomnogram. The recognition of abnormal breathing patterns is important for health monitoring and disease prognosis (LibreTexts, 2018). Generally, abnormal respiratory patterns are induced by injury of respiratory centers, use of narcotic medications and respiratory muscle weakness (Yuan et al., 2013), stroke, heart failure (Lanfranchi et al., 1999), and other conditions.

Recently, there have been several works that perform respiratory pattern classification methods based on contactless sensors including optical sensors and radars. The respiratory patterns can be classified using the respiratory signal extracted from the radar range bin corresponding to the subject's chest wall. The respiratory patterns can be used to implement identity authentication or breathing disorder recognition. Identity authentication is the characteristic analysis of the breathing signal (mainly normal breathing) of specific subjects, while breathing disorder recognition focuses on respiratory pattern classification (mainly abnormal breathing) regardless of the subject. For the identity authentication, Islam et al. (2020) reviewed and summarized the existing radar-based identity authentication algorithms, and evaluated the state-of-the-art identity authentication algorithms' performance and limitations. For the breathing disorder recognition which is related to this study, in Wang et al. (2020a,b), authors implemented the RP classification using recurrent neural networks based on 60-s respiratory signal extracted from a depth camera. It was trained to classify six respiratory patterns (eupnea, tachypnea, bradypnea, Biot's respiration, Cheyne Stokes respiration, and central apnea). There are also several machine-learning-based classification schemes based on 30-s radar-extracted respiratory signals. They were designed to classify respiratory patterns including normal breathing, Biot's respiration, Cheyne Stokes respiration, dysrhythmic breathing in general, Kussmaul breathing and central apnea. In Miao et al. (2017), an SVM-based classifier was developed to classify four breathing patterns with a total accuracy of 90%. In Feng et al. (2019), a KNN-based classifier was developed to classify six breathing patterns with a classification accuracy of each breathing pattern ranging from 60 to 97%. In Zhao et al. (2019), an SVM classifier was developed to classify six breathing patterns with an average accuracy of 94.7%. However, for the radar-based breathing disorder recognition algorithms mentioned above, they were all implemented in a batch processing manner. The reason is that the respiration signal distorted by the random body movement needs to be manually filtered out, otherwise it will affect the classification accuracy (Le Kernec et al., 2019). That is why the existing radar-based breathing disorder recognition methods are not applicable in continuous stream processing.

In our previous work (He et al., 2020), we used Kinect to detect and locate the subjects using 3D skeleton information. This article extends this initial work by implementing a real time system capable of estimating RR and classifying RP for two persons.

## 2. Contribution

The main contributions of this work are as follows

- Many factors can affect respiration rate estimation including small movements, detection of incorrect range bin by the radar, other movements in the room, orientation toward the radar, clutter. If the breathing is irregular, aperiodic or follows some of the breathing patterns detected in this article, it is difficult to determine the radar range bin corresponds to the subject's chest. We have presented a real-time system for robust RR estimation including detection of the chest by using radars and a depth camera, selecting the signal from the radar that has the best “view” of the subject's chest, classifying the respiration patterns, estimating signal quality only in case when the person is stationary and detected pattern is eupnea and then estimating the respiration rate.
- RR estimation and RP classification of multiple people is challenging especially if the people occupy the similar range bins of the radars. Associations of signals from the radars to each monitored person is a challenge as well especially when people move. Our contributions at the system level that allow us to partially address these challenges by utilizing depth camera to do data association and intelligently selecting radars that “see” subjects in different range bins.
- To the best of our knowledge this is the first real-time implementation of the breathing disorder recognition using data from IR-UWB radars.

## 3. Materials and Protocols

**Figure 1:**
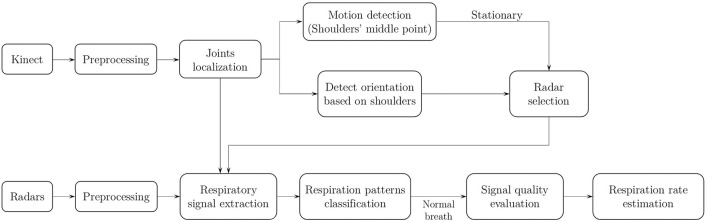
The workflow of RP classification and RR estimation using Kinect camera and IR-UWB radars.

The steps of RP classification and RR estimation using Kinect camera and IR-UWB radars are shown in Figure 1. The data is collected by a Kinect camera, three IR-UWB radars and the respiration belts that are employed as the reference devices for RR measurement. The data pre-processing includes data synchronization, resampling, signal denoising and clutter removal. The subject's localization include conversion of the joints' coordinates (chest, left, and right shoulders) from a rotated Kinect coordinated system to a reference coordinate system. A motion detection algorithm is implemented to determine if a subject is moving or stationary based on the average velocity of the middle point between the shoulders. If a subject is stationary, then a radar selection approach is considered to select the best radar based on the subject's location, orientation, and the distance of the subject's chest from the radar. The respiratory signal will be extracted from the selected radar data and the RP classification algorithm will be applied. RR will be estimated only if the subject is breathing normally and the respiratory signal quality is good. Our work aims to provide a real-time respiration monitoring system that can be used in long-term healthcare monitoring applications, such as home healthcare monitoring, senior apartment and hospitals. In this article, several different experiments were conducted and each module was verified separately, a real time system that includes all of the proposed approaches was also implemented.

### 3.1. Study Population

Five healthy subjects (3 males and 2 females) participated in the single-subject RR monitoring experiments and three of these subjects (2 males and 1 female) participated in the two-subjects RR monitoring experiments. Besides, two healthy subjects (2 males) participated in the single-subject RP classification experiments and 9 different subjects participated in building a dataset for training the RP classification models. The experiment protocol was approved by the University of Ottawa Research Ethics Board.

### 3.2. Experiment Protocol

For one-subject RR experiments, each subject was sitting stationary on an office chair within the radar detection area and breathing normally. The simultaneous radar, reference respiratory signal and Kinect data were collected for 2 min in one experiment. Each subject was asked to perform two sets of experiments with their chests pointing toward radar1 and radar2, respectively. The subject's location and orientation were different for each experiment.

**Figure 2:**
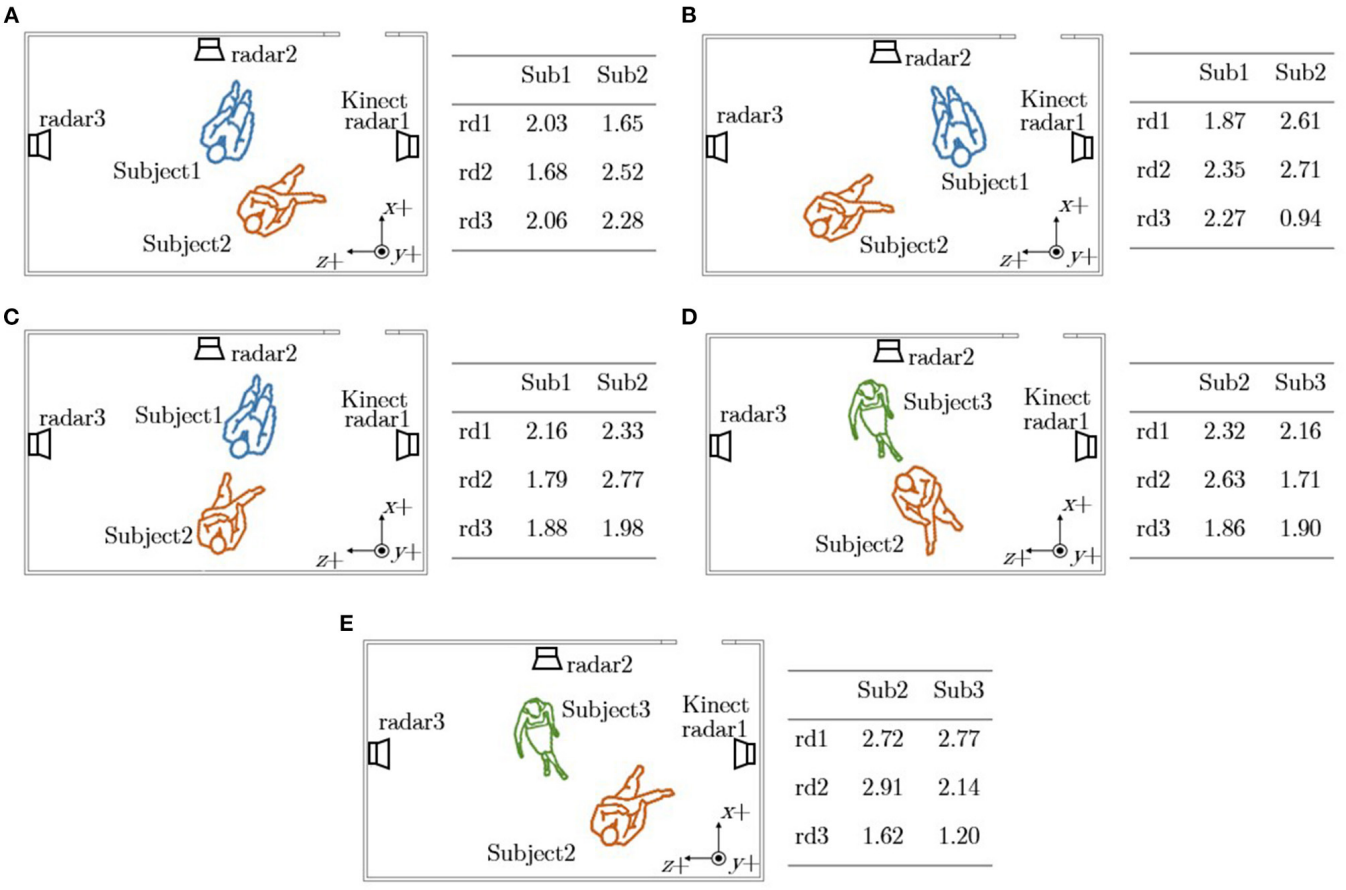
The setup that shows the placement of radars and Kinect for the experiments of two-subjects RR estimation. The position of sensors are the same in all figures while the orientation and subject location changes. The subjects' locations, orientations and the distances between subjects' chests to three radars (unit: meters) in **(A)** Experiment1, **(B)** Experiment2, **(C)** Experiment3, **(D)** Experiment4, and **(E)** Experiment5.

In two-subjects RR experiments, two subjects were sitting stationary on the chairs within the radar detection area. The simultaneous radar, reference respiratory signal and Kinect data were collected for 2 min in each experiment. Five experiments were conducted with different subjects' locations and orientations. The subjects' locations and orientations were set intentionally for each experiment to test our system in realistic situations and the details of each experiment are demonstrated in Figure 2.

For one-subject RP experiments, we collected data separately for training and testing. To construct a respiration pattern signal dataset for model training, 9 healthy human volunteers (6 males and 3 females) were recruited to emulate breathing corresponding to each respiration pattern and used the radar to record their respiration movement. In each experiment, a volunteer was asked to imitate one of the respiration patterns for 1 min under a fixed radar-chest distance of 1.5 m. Each of the volunteers was asked to perform at least 2 sets of experiments. After the signal collection, each 1-min breath data chunk was segmented into four 15-s chunks. The non-stationary data chunks, which include respiration-irrelevant signals and distorted respiratory signals, are also selected from these radar-collected data chunks. We also designed a simple respiratory signal simulator (Han, 2021) according to the characteristic of the radar-collected apnea to augment the classes with less data.

For testing, the subjects were asked to imitate certain respiration patterns at a certain stage of the experiment. The experiment was conducted in the following manner: the subject was asked to perform eupnea and imitate Cheyne Stokes respiration, Kussmaul respiration and apnea in the monitored area. Except for the period of simulating the respiration patterns, the subject was able to move freely. Two healthy male subjects participated in this experiment. None of these two subjects participated in the previous experiment used to build respiration pattern dataset to prevent any bias in classification. The experiment duration was determined according to the subject's physical status. The experiment duration for the first subject was 5 min and for the second subject was 8 min. All collected data were manually labeled after the experiment.

The two-subject RP classification experiments are not presented in this article. The experiment results are similar to those of the one-subject RP experiments. The only difference in results is observed when two subjects appear in the same range bin of the radar - then the extracted respiratory signal is corrupted. This problem is resolved by selecting one of the three radars for which the distance between the subjects and the radar is different and therefore the extracted respiratory signals are in different range bins.

### 3.3. Signal Acquisition System

The signal acquisition system consists of three IR-UWB radars, a Kinect camera and two respiration belts. The UWB radar used in this experiment is a Xethru^TM^ X4M03 development kit manufactured by Novelda (Oslo, Norway), which uses the X4 chip operating at a center frequency of 7.29 GHz. The radar is equipped with 6-8.5 GHz directional patch antennas with a beam of 60° in both azimuth and elevation axes. The sampling rate of this radar is 23 GS/s in fast time, up to 20S/s in slow time (Baird, 2017) and it was set to 17 S/s in this work. The output data is in the form of a matrix where rows represent observations or samples in slow time and the columns represent samples in fast time (corresponding to range bins). The range resolution of the X4 radar is approximately 5.22 cm. During experiments the radar was programmed to record data up to a distance of 9.4 m (XPengZhao, 2020) away from the radar which resulted in 180 columns in the data matrix. These UWB radar sensors were placed at different locations in the laboratory, as shown in **Figure 4A**. Since the radar used in this study is pseudo-random noise UWB radar, each radar has its unique pattern when transmitting. Pulses from other radar will be treated as noise and therefore the radar pulses from different radars would not interfere with each other (Andersen et al., 2017; Novelda, 2018).

**Figure 3:**
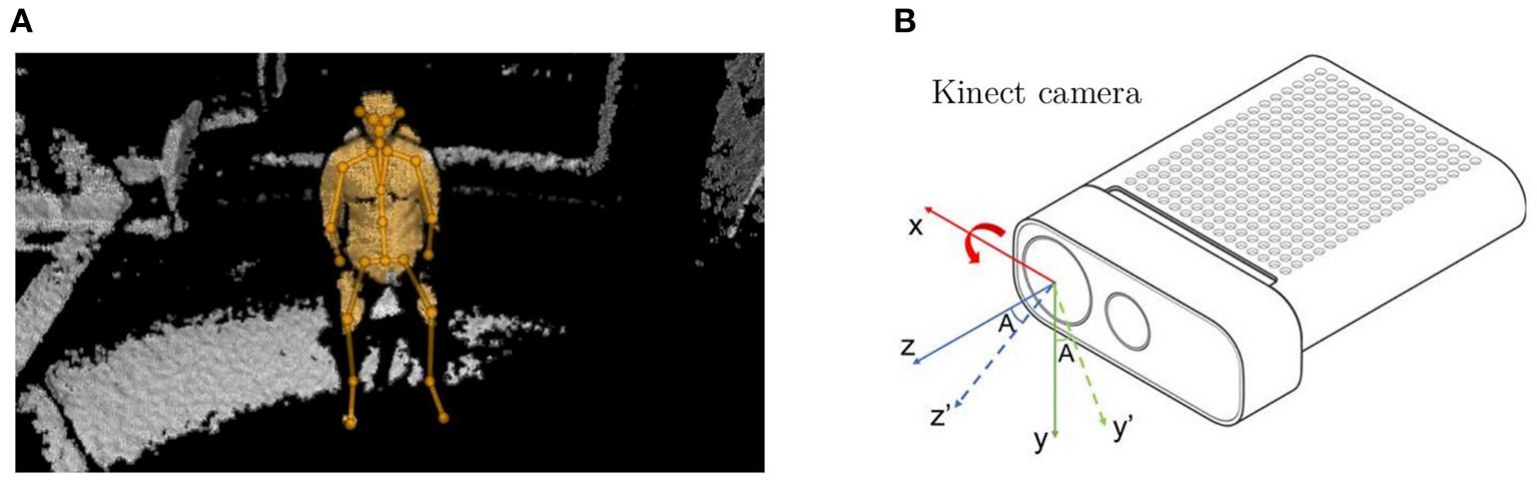
**(A)** Joints and skeletons detected and visualized by the Azure Kinect Tracking Viewer when a human subject was sitting on a chair. **(B)** The Kinect frame is rotated about x-axis by an angle A (solid lines: reference frame; dash lines: rotated frame).

**Figure 4:**
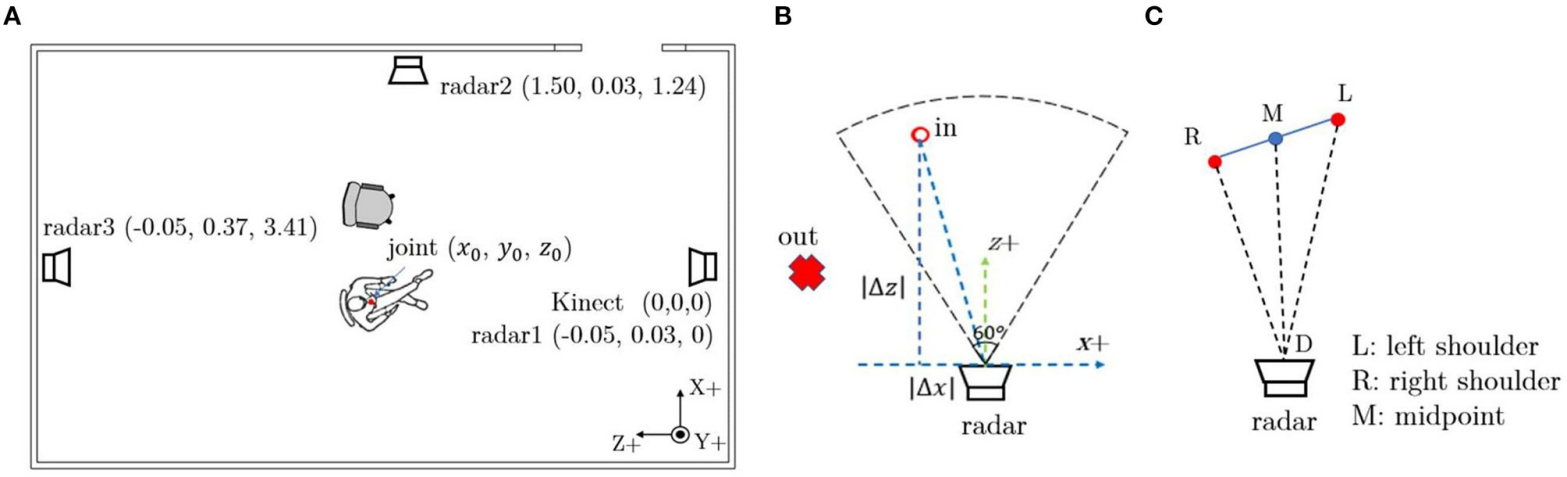
**(A)** The location of the devices and their coordinates in the reference frame. **(B)** The method to determine if a subject is within radar detection area (black dash line: radar detection area). **(C)** Radar selection method based on subject's orientation.

The Azure Kinect (Microsoft®) is an RGB-D camera that includes a 12 megapixel RGB camera supplemented by a 1-Megapixel Time-of-Flight (ToF) depth camera. The Kinect body tracking software can detect and track multiple humans simultaneously and the maximum detection range of the depth camera is 5.46 m (Microsoft, 2021). Each detected human is assigned an ID for temporal correlation between frames and the kinematic skeleton. A skeleton includes 32 joints and all joint coordinates are given relative to the depth camera 3D coordinate system (Microsoft, 2019a). An example of joints detected by the Kinect camera when a subject was sitting on an office chair is shown in Figure 3A. This work only extracts the coordinates of three joints (chest, left shoulder, right shoulder) from Azure Kinect Body Tracking SDK and does not use RGB or depth frames due to privacy concerns. The Kinect camera operated at 1 fps in this work and it was placed on a 2.11 m pole. The experimental setup for this work is shown in Figure 4A.

There are two Go Direct®respiration belts (Vernier) employed to measure reference respiratory waveform from the subjects' chest. The reference RR belt is a force sensor based device which can measure the movement of the chest caused by respiration and the sampling rate is 10 Hz.

A laptop (Intel Core i7, Nvidia GeForce GTX 1050 Ti, 16 GB RAM, Windows10) was used for data collection, data storage and analysis. Our real-time system was also implemented on this laptop. Data was saved in separate CSV files for analysis, the signals were processed and the proposed algorithms were implemented using Python 3.5.

## 4. Methods

### 4.1. Data Pre-processing

All the signals were resampled to 17 Hz because of different sampling frequencies of the radars and breathing belts. The background noise was removed from radar signals by subtracting the background data from raw radar data. Then the singular value decomposition (SVD) was applied to filter the clutter from radar signals.

### 4.2. Subject's Localization

The Kinect camera was employed for body tracking and localization. As defined by the manufacturer, the origin [0, 0, 0] is located at the focal point of the camera, and the reference coordinate system is oriented such that the positive x-axis points to the right, the positive y-axis points down and the positive z-axis points forward (Microsoft, 2019b), as shown in Figure 3B (solid lines). In practice, to cover a larger detection area, the Kinect camera was placed on a pole pointing downwards at a certain angle that means the reference frame of the Kinect was rotated about its x-axis by that angle as shown in Figure 3B (dash lines). The rotation matrix widely used in robot kinematics was applied to transform the coordinates from the rotated frame to the reference frame. Since the Kinect coordinate system was only rotated about x-axis in this work, the joints' coordinates of the subject in the reference frame can be converted from the rotated frame by calculating the product of the rotation matrix and the detected coordinates:


(1) $${}_{}^{0}P{=}_{}^{0}R_{1}^{}\cdot_{}^{1}P=\left[\begin{array}{ccc} 1 & 0 & 0\\ 0 & \cos A & -\sin A\\ 0 & \sin A & \cos A \end{array}\right]\;\cdot \;\left[\begin{array}{c} x_{rot}\\ y_{rot}\\ z_{rot} \end{array}\right]$$

where, ${}^{0}P=\left[x_{0},y_{0},z_{0}\right]$ are the coordinates of one joint in the reference Kinect frame, ${}^{0}R_{1}$ is the rotation matrix and *A* is the rotation angle which is -30° in this study, ${}^{1}P=\left[x_{rot},y_{rot},z_{rot}\right]$ are the coordinates of that joint in the rotated Kinect frame. Then the Euclidean distances between subject's joints and each radar can be calculated as:


(2) $$\begin{array}{r} dist\left(i\right)=\sqrt{{\left(x_{0}-x_{i}\right)}^{2}+{\left(y_{0}-y_{i}\right)}^{2}+{\left(z_{0}-z_{i}\right)}^{2}}\;\left(i=1,2,3\right) \end{array}$$

where, *dist*(*i*) is the distance between the subject's joint to each radar, [*x*_0_, *y*_0_, *z*_0_] is the joint coordinates in the reference Kinect frame and [*x*_*i*_, *y*_*i*_, *z*_*i*_](*i* = 1, 2, 3) are the radar's coordinates in the reference Kinect frame. The coordinates of the Kinect and three radars in the reference coordinate system can be found in Figure 4A.

### 4.3. Motion Detection

After locating a subject using a Kinect camera and the proposed method, a threshold based motion detection algorithm was developed to determine if a subject is moving or stationary. The middle point of the left and right shoulders was used as a reference point to calculate the movement trajectory. A subject is determined non-stationary (or moving) if the average velocity of the reference point is greater than a threshold of 0.7 m/s which is similar to the slow walking speed. The RR estimation and RP classification will only be conducted if the subject is stationary.

### 4.4. Radar Selection

**Figure 5:**
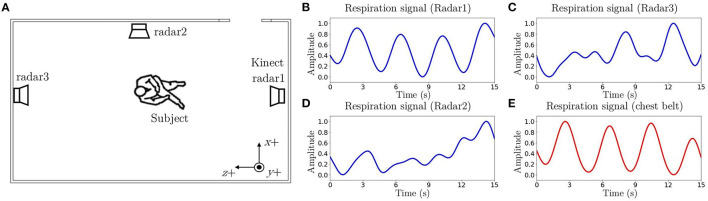
An example of the relation between subject's orientation and respiration signals. **(A)** A subject was sitting on a chair facing toward Radar1. **(B)** The respiration signal extracted from Radar1 (*RR*_*est*_ = 15.45 breaths/min). **(C)** The respiration signal extracted from Radar2. **(D)** The respiration signal extracted from Radar3. **(E)** The respiration signal detected by a reference breathing belt (*RR*_*ref*_ = 15.52 breaths/min).

A single radar has limited detection area and therefore three radars were placed at different locations to cover the entire room. A good quality respiratory waveform can be extracted when the subject is facing toward the radar. A demonstration of the relation between subject's orientation and extracted respiration signals from three radars can be seen in Figure 5. The respiration signal detected by radar1 (Figure 5B) is almost the same to the respiration signal measured by a breathing belt (Figure 5E) when a subject was sitting on a chair and facing toward radar1. Therefore, it is necessary to include a radar selection algorithm based on the subject's location and orientation to extract the best possible respiratory waveform for RP classification and RR estimation.

In this work, the beam solid angle of the employed radar is 60°. As shown in Figure 4B, a subject is determined in the radar detection area (beam solid angle) if 60° < arctan(|Δ*z*|/|Δ*x*|) ≤ 90° and *dist* < 5*m* (calculated using Equation 2). |Δ*x*| is the absolute difference between the subject's chest coordinates and radar's coordinates on the x-axis and |Δ*z*| means the absolute difference between the subject's chest coordinates and radar's coordinates on z-axis.

If a subject is detected by multiple radars, then the radar toward which the subject is oriented will be selected. Ideally, if a subject is facing straight to the radar, the ∠*RMD* should be 90° (see Figure 4B). The orientation angle ∠*RMD* can be expressed as:


(3) $$\begin{array}{r} ∠RMD=\arccos\frac{RM^{2}+MD^{2}-RD^{2}}{2\cdot RM\cdot MD} \end{array}$$

where RM is the distance between right shoulder to the shoulders' middle point on *xz* plane (Kinect reference frame), MD is the distance between the shoulders' middle point and the radar on *xz* plane, RD is the distance between the right shoulder and the radar on *xz* plane as shown in Figure 4C. If a subject is in multiple radars' detection areas, then the orientation angle ∠*RMD* will be calculated for each of them and the radar whose orientation angle is closest to 90° will be selected.

If there are multiple subjects detected, an algorithm was developed to select the corresponding radar for each subject based on their orientations and locations. The pseudocode of the proposed radar selection method can be found in Supplementary Algorithm 1. This radar selection algorithm is demonstrated with several examples.

- If the difference of the distances between chests of the subjects and the radars is greater than 30 cm (6 range bins), then the radars they are oriented to will be selected for respiratory signals extraction. Such experiments are Experiment 1-4 in Section 3.2.
- If the difference of the distances between subjects' chests and the radars is smaller than 30 cm (6 range bins), then another radar will be selected for estimating RR. For example, in Experiment5 in Section 3.2, it is estimated that Subject1 and Subject2 are oriented toward radar1 and radar2, respectively. However, the difference of the distances between the chests of Subject1 and Subject2 to radar1 is around 5 cm which means that it is difficult to identify Subject1's respiratory signal using radar1. Therefore the radar whose orientation angle is the second closest to 90° will be selected for Subject1. This means that radar2 will be selected for both Subject1 and Subject2. Also, the difference of the distances between subjects' chests to radar2 is greater than 30 cm which makes it possible to extract respiratory signals from radar2 for both subjects.

### 4.5. Respiratory Signal Extraction

If the subject is detected stationary and a radar is selected, the respiratory signal will be extracted. A narrow range of ±3 range bins (similar to the thickness of the human body) around the calculated distance between the subject's chest and the radar using Equation (2) is extracted from the selected radar data matrix. Within these range bins, the one with the maximum variance is determined as the respiratory signal.

### 4.6. Respiratory Pattern Classification

**Figure 6:**
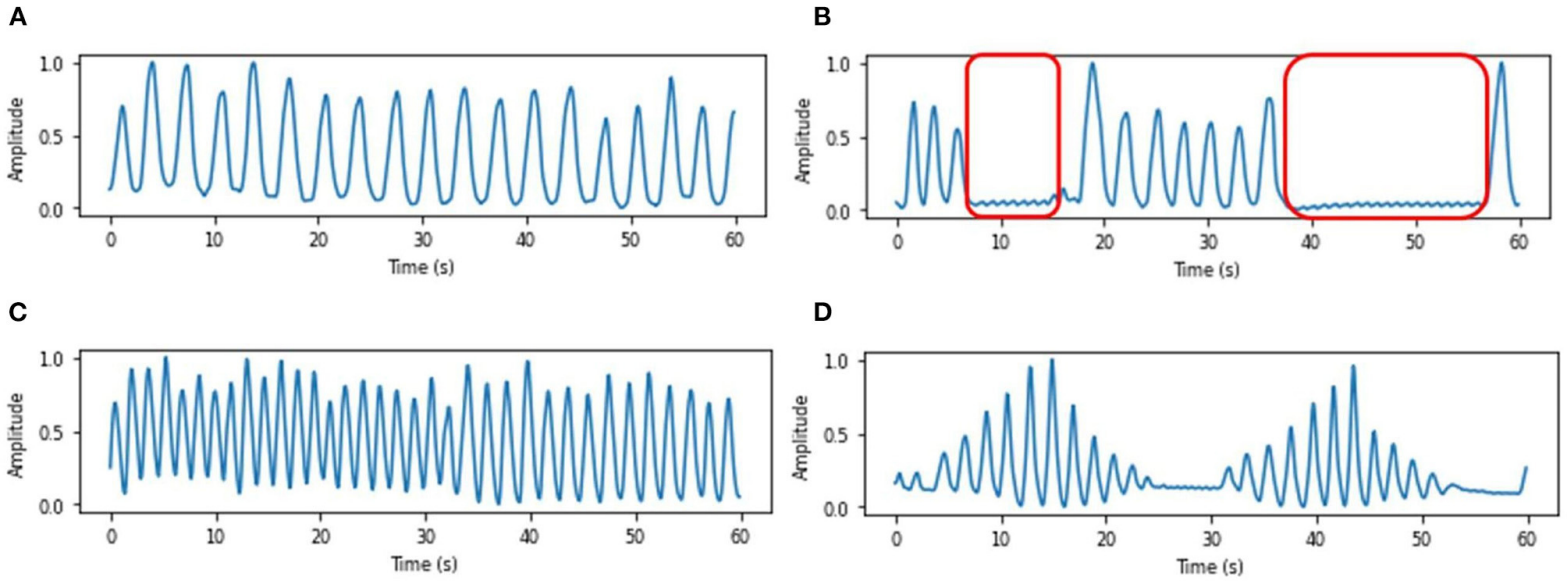
Respiration belt samples of typical respiratory patterns, **(A)** Eupnea, **(B)** Apnea, where red boxes represent the apnea events, **(C)** Kussmaul respiration, and **(D)** Cheyne Stokes respiration.

RP classification includes two steps: classification itself and pattern discrimination. The second step is needed in order to prevent miss-classifications due corrupted signal. Pattern discrimination further processes the signal waveform to determine signal quality, detect individual breaths, and also confirms respiration patterns. The extracted breath sample is classified into five patterns: eupnea (normal breathing), Cheyne Stokes respiration (CSR), Kussmaul respiration, apnea, and non-stationary. The pattern “non-stationary” is used to classify the extracted respiratory signals which are distorted by minor body movements, or the respiration-irrelevant signals. If the subject localization module does not output a correct radar-chest distance, the system may extract the signal from a range bin irrelevant to respiration chest movement, which we refer to as the respiration-irrelevant signal. The distorted respiratory signals and respiration-irrelevant signals would deeply influence the classification accuracy as shown in Le Kernec et al. (2019). The illustrations of some typical respiratory patterns are shown in Figure 6.

A machine learning classifier based on three types of statistic features: time-domain features, time-frequency domain features and short-term energy features as shown in Table 1 (Zhao et al., 2019) was developed. For the time-frequency domain features, the instantaneous frequency is calculated by the Hilbert Transform. For the classification, three types of classifiers including support vector machine (SVM), decision trees (DT), and Random Forest (RF) were implemented using the Python scikit-learn library. To find out the best performance of each classifier, several parameters of each classifier have been tested, as shown in Table 2. The parameters which are not mentioned in the table were set as scikit-learn default values.

**Table 1:** Statistical features.

**Categories**	**Features**
Time domain features (signal peak)	Amplitude variability
	Mean amplitude
	Maximum amplitude
	Number of peaks
Time-frequency domain features (instantaneous frequency)	Variability
	Mean
	Maximum
	Minimum
	Range
Short-term domain features (signal energy for 5-s segment)	Variability
	Maximum
	Minimum
	Range

**Table 2:** Parameter settings of the classification models.

**Classifier**	**Parameters**
Support Vector Machine	Kernels: “linear”, “polynominal”, “Gaussian”, “sigmoid”
Decision Trees	Max depth: 5, 10, 100
Random Forest	Number of trees: 10, 100, 1,000

**Figure 7:**
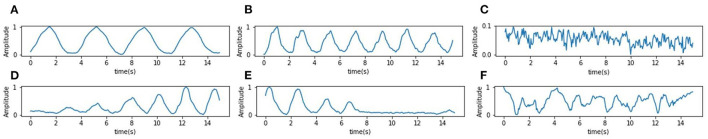
Respiratory pattern database examples of each pattern, **(A)** Eupnea, **(B)** Kussmaul respiration, **(C)** Apnea, **(D)** CSR (crescendo phase), **(E)** CSR (decrescendo phase), and **(F)** Non-stationary.

The details of the training dataset is described in Section 3.2. We have mentioned that collected data was augmented with simulated data so that the database contains 250 sets of 15-s breath chunks, including 50 sets of eupnea, 50 sets of CSR, 50 sets of Kussmaul respiration, 50 sets of apnea and 50 sets of non-stationary chunks. The 15-s chunks of each pattern are shown in Figure 7. Since the period of CSR is normally longer than 15 s, a full period of CSR is demonstrated in Figures 7D,E representing the crescendo phase and decrescendo phase, respectively.

All the classifiers listed in Table 2 were tested with the database by 10-fold cross-validation. The RF classifier with 100 trees achieved the highest accuracy of 90%, the SVM classifier with linear kernel achieved the highest accuracy of 86% among the SVM models and the DT classifier with max depth of 10 achieved the highest accuracy of 85% among the DT models. Thus, the RF classifier with 100 trees was selected as the classifier of the RP classification model.

We developed a breathing pattern discriminator to further analyze each breath chunk and possibly modifies the results obtained by the classifier. The pattern discriminator is developed based on the morphology of the breathing signal. It comprises two sections: signal quality evaluation section and specific pattern evaluation section. The signal quality evaluation criteria are proposed based on the signal quality index (SQI) algorithm that consists of the following steps (Charlton et al., 2021):

- A median filter with a kernel size of 5 is applied to filter the original signal. This filtering process is used to improve the signal-to-noise ratio (SNR) for the signal quality evaluation.
- Then, the signal is normalized to a range of [0,1].
- The peaks and troughs (local extrema) in the signal chunk are detected. The extrema are defined according to the criterion that the normalized peak and trough should have a prominence larger than 0.15.
- The chunks which have at least one relevant trough between the time spanning consecutive relevant peaks are defined as valid breaths.

At the second stage, the respiratory signal plausibility is assessed based on the valid respiratory interval. Respiratory signals with less variable cycle time have higher plausibility. Two criteria were used: (i) the normalized standard deviation of breath intervals should be smaller than 0.25, which is used to permit only moderate variation in the duration of detected breaths; (ii) there are at least 3 valid breath intervals in a 15-s chunk. Any chunk which did not satisfy these criteria was deemed to be of low quality and eventually labeled as “non-stationary”. Only the respiratory chunks which satisfy the criteria can be considered high quality and retain their label given by the classifier.

**Figure 8:**
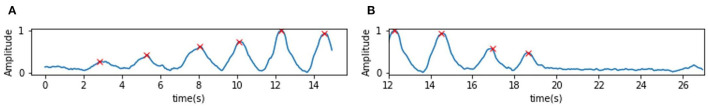
Pattern evaluation example **(A)** CSR crescendo phase **(B)** CSR decrescendo phase.

The pattern evaluation is mainly designed for the identification of CSR. It is developed based on the tidal volume variation feature of crescendo and decrescendo in CSR, as shown in Figure 8. Firstly, we detect all the peaks in the signal, and locate the peak with maximum value, such as the peak around 12 s in Figure 8. We set this location as a boundary, and compare the amplitude of each peak to see whether they meet the following criteria: the amplitude of each peak precede the boundary is smaller than the latter peak, as Figure 8A, and the amplitude of each peak after the boundary is smaller than the former peak as Figure 8B. Only the breath chunks which are not only labeled as CSR by the classifier but also meet the criteria of both sections would be confirmed as CSR. A chunk with CSR label that passes the signal quality evaluation, but fails to pass the CSR pattern evaluation would be eventually labeled as eupnea.

### 4.7. Respiration Rate Estimation

After detecting the normal breathing signals, a 3*rd* Butterworth bandpass filter with the lower cutoff frequency of 0.1 Hz and higher cutoff frequency of 0.5 Hz (corresponding to 6–30 breaths/min) was applied to further denoise the signal, and a peak interval based approach was applied for RR estimation. The peaks of the respiration signal has already been detected in the last section, the mean peak-to-peak interval Δ*t* between every two successive respiration pulses is calculated and the estimated RR can be expressed by Equation 4


(4) $$\begin{array}{r} RR_{est}=60/\Delta t \end{array}$$

The reference RR (*RR*_*ref*_), when the respiration signal is recorded by the breathing belt, is also calculated using this approach.

## 5. Results

### 5.1. Subject Tracking and Localization

An experiment was conducted to evaluate the performance of the Kinect camera and the proposed approach of subject localization. We conducted an experiment in which the subject was tracked using Kinect around the room and the outcomes were compared to a reference position. (please see Supplementary Figure 1). Since the human body is a three-dimensional form and the body may swing during the test. We find that the Kinect and the proposed algorithm can accurately locate human subjects. However, the location of the chest can have an error of several tens of cm. Since the range bin of the radar is about 5 cm, we perform additional range bin selection in order to detect the range bin where the breathing signal is the strongest for each radar as explained before.

### 5.2. Respiration Pattern Classification

The experiment for RP classification was described in Section 3.2. Data was collected as well as processed in real-time. In the pre-processing stage, the data is segmented into 15-s chunks with 12-s overlap. Eventually, there were 103 breath chunks collected from the first subject and 151 breath chunks from the second subject, a total of 254 chunks. The respiratory pattern classification was implemented in real time.

**Figure 9:**
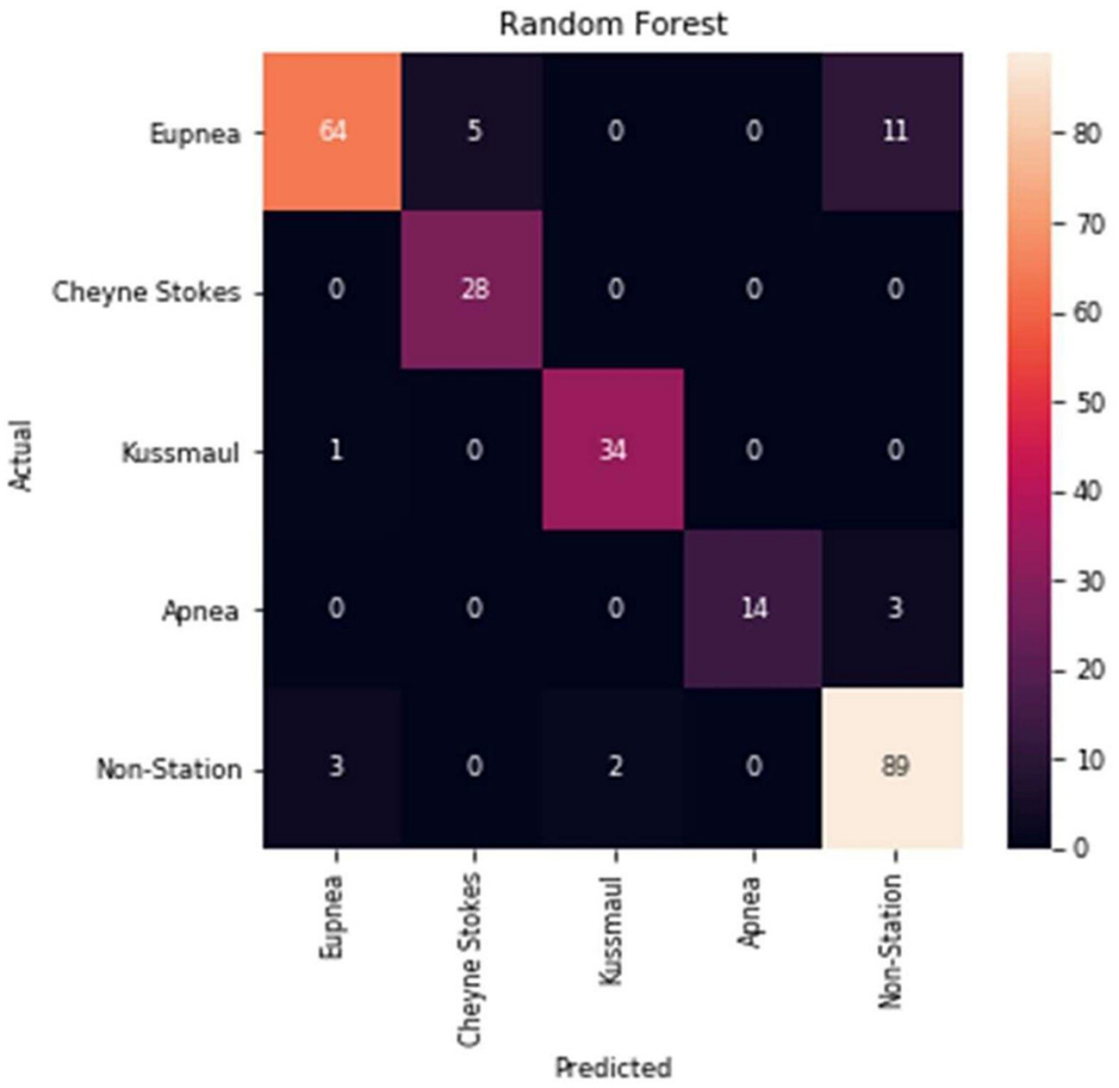
Confusion matrix of the classification model.

Table 3 and Figure 9 summarizes the model performance of the classification. The average classification time for each sample is 0.008 s. According to the results, the model composed of a machine learning classifier and a pattern discriminator is able to implement accurate RP classification.

**Table 3:** Classification report of statistic feature-based model.

	**Precision**	**Recall**	**F1-score**	**Support**
Eupnea	0.94	0.8	0.86	84
CSR	0.85	1	0.92	32
Kussmaul	0.94	0.97	0.96	34
Apnea	1	0.82	0.90	11
Non-stationary	0.86	0.95	0.90	93

### 5.3. Respiration Rate Estimation

Experiments were conducted either with one subject or two subjects to assess the performance of the proposed algorithms for RR estimation. The experiments were described in Section 3.2.

The synchronized data from all the sensors was divided into 15 s chunk with 12 s overlapping for RR analysis. The RR estimation error for the *i*^*th*^ chunk is a function of the absolute error between the estimated *RR*_*est*_ and corresponding reference measurement *RR*_*ref*_ which is extracted from a breathing belt (*eRR*(*i*) = |*RR*_*est*_(*i*) − *RR*_*ref*_(*i*)|). The RR estimation error was validated using two statistical metrics; mean of the RR estimation error *M*_*eRR*_ and standard deviation of the RR estimation error *SD*_*eRR*_.

#### 5.3.1. RR Estimation for One Subject

The mean and standard deviation of the RR estimation error for each subject can be found in Table 4. The correlation plot and Bland-Altman plot of the estimated RR and reference RR for one-subject experiments can be found in Supplementary Figure 2A.

**Table 4:** Mean and standard deviation of the RR estimation error between the estimated RR and the reference RR of one-subject experiments (unit:breaths/min).

	**Subject1**		**Subject2**		**Subject3**		**Subject4**		**Subject5**	
	***M*_*eRR*_**	***SD*_*eRR*_**	***M*_*eRR*_**	***SD*_*eRR*_**	***M*_*eRR*_**	***SD*_*eRR*_**	***M*_*eRR*_**	***SD*_*eRR*_**	***M*_*eRR*_**	***SD*_*eRR*_**
Experiment 1	0.20	0.19	1.92	0.99	0.34	0.37	0.30	0.40	0.55	0.25
Experiment 2	0.59	0.40	1.01	0.99	0.22	0.38	0.69	0.46	0.27	0.29

#### 5.3.2. RR Estimation for Two-Subjects Experiments

The radar with the best monitoring angle selected by the proposed method, the mean and standard deviation of the RR estimation error of each subject in each experiment are shown in Table 5. The correlation plot and Bland-Altman plot of the estimated RR and reference RR for two-subjects experiments can be found in Supplementary Figure 2B.

**Table 5:** The selected radar, mean and standard deviation of the RR estimation error between the estimated RR and the reference RR of two-subject experiments (unit:breaths/min).

	**Subject1**			**Subject2**			**Subject3**		
	**Selected radar**	***M*_*eRR*_**	***SD*_*eRR*_**	**Selected radar**	***M*_*eRR*_**	***SD*_*eRR*_**	**Selected radar**	***M*_*eRR*_**	***SD*_*eRR*_**
Experiment 1	radar2	0.89	0.92	radar1	0.30	0.26	–	–	–
Experiment 2	radar2	0.71	0.56	radar1	0.50	0.46	–	–	–
Experiment 3	radar2	0.67	0.47	radar2	0.48	0.34	–	–	–
Experiment 4	–	–	–	radar2	1.15	0.92	radar2	0.59	0.45
Experiment 5	–	–	–	radar2	0.81	0.74	radar2	0.74	0.57

## 6. Near Real-Time Implementation

In this work, we implemented the software that can stream and process multiple sensors in near real time (NRT) and shows results on a graphical user interface (GUI). An NRT solution is a software application that can consume, process, and generate results very close to real-time but can not be guaranteed that all the processing will be completed before the deadline (Saxena and Gupta, 2017).

**Figure 10:**
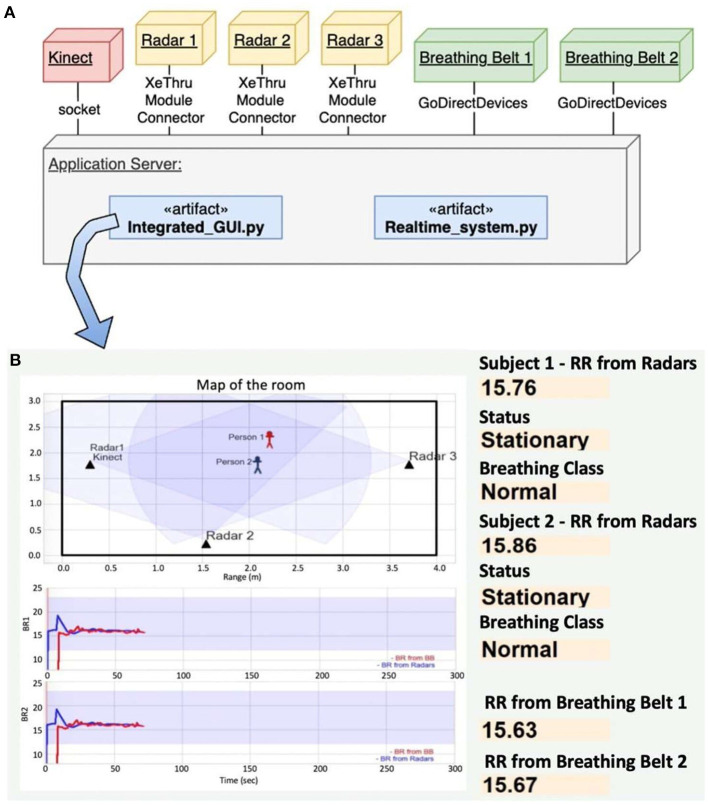
**(A)** Deployment diagram of our near real-time system, **(B)** GUI that shows the estimated location of two subjects on the top left, reference (blue) and radar-estimated (red) RRs for subject 1 (graph in the middle and subject 2 (graph on the bottom), and the estimated RRs and patterns for the current 15 s windows on the right.

Our NRT implementation was performed using multiprocessing and a fixed-length overlapping sliding window. The data acquisition from multiple devices (Kinect and Radars) requires multiprocessing framework. We have used the multiprocessing python package that supports spawning processes using an API similar to the threading module (McKerns et al., 2012). In the system, new processes are spawned and executed simultaneously to communicate with the devices and the communication between these processes is implemented using queues. The deployment diagram, shown in Figure 10, gives a general overview of the hardware/devices in the system, the links of communication between them, and the placement of software files in the system. The application server manages all the connection with devices using the software (realtime_system.py) that was implemented in Python. The application server is a machine with an Intel Core i7-7700HQ, 16GB RAM and Windows 10 Enterprise. Every device supports a specific type of connection. The communication with Kinect was performed using sockets, and with the Radars and Breathing belts *via* specific APIs provided by the device manufactures, see the Figure 10A.

The software artifact hosted in the application server is responsible for the real-time respiration monitoring and classification tasks using the raw data that come from RGB-D camera and IR-UWB radars. This is performed using fixed-length overlapping sliding window (Dehghani et al., 2019; Malmir and Rezvani, 2019). The sliding window process is defined as follows. First we define a window/chunk size *T* of 15 s duration and an overlap *m* of 12 s duration. Following that, the first window *X*_1_ is located in the interval [0, *T*], the second window *X*_2_ is located in interval [*T* − *m*, 2*T* − *m*], and similarly, the *n*^*th*^ window lies in interval of [(*n* − 1)(*T* − *m*), *nT* − (*n* − 1)*m*]. The time complexity of this approach is *O*(*n*) and it allows us to perform the respiration monitoring and classification tasks with a time span of about 3 s with initial delay of 15 s. The total latency of the system is determined by the data window size 15 s and data processing latency. A chunk of 255 elements (15s × 17 samples/s) is used to compute the RR, Subject Status and Breathing Class. The results are displayed on the GUI hosted in the application server, see the Figure 10B. The GUI displays the location of subjects on the map, extracted and reference respiratory waveform, estimated RR (radar) and reference RR (belt), the status of the subject (moving or stationary) and the breathing class.

## 7. Discussion and Conclusion

In this study, a system comprised of an RGB-D camera and three IR-UWB radars was developed for remote RP classification and RR estimation. The system of this kind has a potential to classify posture, activities, detect falls, estimate breathing rate and heart rate and further learn about behavior and patterns of monitored subjects. We worked on several of these applications in our previous works using radars: fall detection (Sadreazami et al., 2019), activities (Valdes et al., 2018), and classification of posture (Baird et al., 2018). All of these works, as well as majority of other published works, focused on a single subject monitoring. In this work, we address the problem of RR estimation and RP classification of two subjects.

Contactless monitoring of multiple people in a confined space has a number of challenges including the inability to detect and/or extract their respiration signals when they are really close to one another, associate the extracted breathing signals to the correct person especially when they move, achieving real-time processing as latency increases with each additional person in the room and so on. There have been prior studies to monitor the respiration of two or more people (Yang et al., 2019; Shang et al., 2020). In Shang et al. (2020), custom radar is designed that supports detection and estimation of RR people. In Yang et al. (2019), multiple people can be detected by a single UWB if they are in the range bins that are 15 cm apart. However, subjects can be in the same range bin (same distance from the radar) even if they are not close to one another and in this case, it will not be possible to estimate their breathing without radar or without using multiple radars.

We observed that even small movements can cause large disturbances of the respiratory signal and walking of one person can significantly affect the respiratory signal of a stationary person being monitored using radars. Kinect is able to detect and track each person in the room but the problem of occlusions among targets still exist with Kinect camera due to which association of respiratory signal to its correct target without mixing becomes difficult. RGB cameras could be used for data association – however we did not consider RGB cameras for privacy reasons. Therefore, we included the depth sensor that detects and track people.

4D imaging and multiple input multiple output (MIMO) beamforming radars could be used for this application. In a recent work, authors have used MIMO radar with a 2D digital beamforming for simultaneous multi-target vital sign monitoring (Feng et al., 2021). The 2D digital beamforming helps finding the subject's chest location and forming individual narrow beams to measure respiration and heart rate accurately.

We decided to use UWB radars as they are compact in size, very simple to use and are cost-effective. The proposed approach assume the relative positions and orientations of the radar systems are known in advance that might not be possible in all the scenarios.

There have been several studies conducted to implement RP classification based on radar collected data; however, they cannot be implemented in real-time since the respiration signal distorted by body movement needs to be manually removed before the classification. However, the respiration signal distortion is an unavoidable challenge in vital signs monitoring through radar. Even though some body movement removal algorithms have been developed (Lazaro et al., 2014; Khan and Cho, 2017), they can only be applied in scenarios that abnormal respiration patterns such as Cheyne Stokes respiration (CSR) are not considered. With those methods, CSR chunks would probably be removed because the respiration signal looks irregular. In this study, we combined the respiration pattern classification with signal morphology analysis to develop an algorithm that can classify the abnormal respiration patterns and body movement. Based on this algorithm, we realized the real-time respiration pattern classification. Classification precision of eupnea, CSR and Kussmaul respiration is 94, 85, 94%, respectively. State-of-the-art RP classification research (Zhao et al., 2019) reported precision of 89, 87, 91% for the same classes. These results depend on the number of subjects, data quality, the number of data points in different classes and so on and therefore it is not appropriate to compare exact values of precision and accuracy of different methods without taking all these aspects into account. However, we can observe that the performance of our classification model in real-time implementation is at the similar level as the performance of the state-of-the-art study.

We also showed that the MAE between the estimated RR and the reference RR for a single subject varies from 0.2 to 1.92 breaths/min and the MAE between the estimated RR and the reference RR for multiple subjects varies from 0.3 to 1.15 breaths/min.

The whole system is implemented in software efficiently so that it monitored two subjects in real-time. It completes processing and presents the results on the GUI with a time span of ≈ 18 s latency, because it relies on data window/chunk size of 15 s and data processing latency of about 3 s. Processing latency could be reduced by using a more powerful CPU to process the data.

In future, we intend to improve the several aspects of the system. We will use 4D imaging radars which might remove the need for using the depth camera. We also plan to develop algorithms for sensor fusion of radar signals instead of performing radar selection. We intend that this article will be a stepping stone toward providing a non-invasive means of monitoring congestive heart failure patients after discharging them from the hospital.

We also intend to implement the respiratory pattern classification to detect respiration patterns related to COVID-19. Radars are very useful for COVID-19 because they are contactless, can be used to estimate distance among people which is important for COVID-19 distancing protocols and to estimate RR and detect RP (Islam et al., 2021). According to Wang et al. (2020a), tachypnea is one of the symptoms of COVID-19 that can be detected with our system.

## Data Availability Statement

The original contributions presented in the study are included in the article/Supplementary Material, further inquiries can be directed to the corresponding author/s.

## Ethics Statement

All procedures performed in studies involving human participants were in accordance with the ethical standards of the Office of Research Ethics and Integrity at the University of Ottawa. Informed consent was obtained from all individual participants involved in the study.

## Author Contributions

SH, ZH, CI, VM, and MB: conceptualization, methodology, programming, and writing—original draft preparation. SH, ZH, and CI: validation. SH, ZH, VM, and CI: data collection. VM and MB: writing—review and edit. MB: supervision and project administration. All authors have read and agreed to the published version of manuscript.

## Funding

This study received funding from Aerosystems International Inc., Montreal, Canada. The funder was not involved in the study design, collection, analysis, interpretation of data, the writing of this article or the decision to submit it for publication. We are also thankful to Natural Sciences and Engineering Research Council of Canada (NSERC) for partially funding this work through MB's Discovery Grant (RGPIN-2020-04417).

## Conflict of Interest

The authors declare that the research was conducted in the absence of any commercial or financial relationships that could be construed as a potential conflict of interest.

## Publisher's Note

All claims expressed in this article are solely those of the authors and do not necessarily represent those of their affiliated organizations, or those of the publisher, the editors and the reviewers. Any product that may be evaluated in this article, or claim that may be made by its manufacturer, is not guaranteed or endorsed by the publisher.
