# Supplementary material for: A Real-Time Respiration Monitoring and Classification System Using a Depth Camera and Radars
Source: Front Physiol. 2022 Mar 9;13:799621. doi: 10.3389/fphys.2022.799621 (PMC8959759; doi:10.3389/fphys.2022.799621)
Supplement: Supplementary file 1 [file Data_Sheet_1.PDF]

A Real-Time Respiration Monitoring and Classification System using a Depth Camera and Radars: Supplementary Material

1 SUPPLEMENTARY ALGORITHM

The pseudocode of the proposed radar selection algorithm is shown in Algorithm 1.

2 SUPPLEMENTARY TABLES AND FIGURES

2.1 Subject localization

Experiment setup: Nine landmarks (gray crosses in Figure S1) were placed on the ground and a subject stood still on each cross for approximately 30 seconds. The coordinates of the middle points of the right and left shoulders (blue crosses in Figure S1) were converted to the Kinect reference frame and the projections on the *xz* plane of the Kinect reference frame are shown in Figure S1.

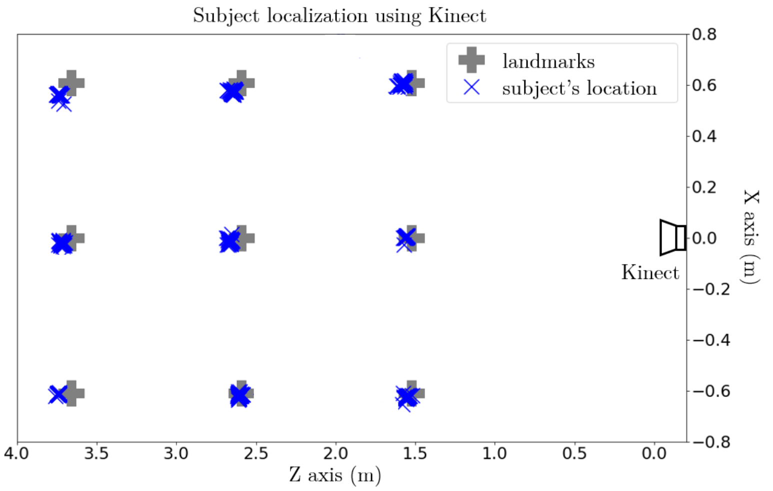

Figure S1. An experiment of subject localization using Kinect depth camera.

2.2 RR estimation

The correlation plots and Bland-Altman plots of the estimated RR and the reference RR for one-subject experiments and two-subjects experiments are shown in Figure S2.

3 SUPPLEMENTARY VIDEO

Supplementary video is attached. The goal of the video is to show our real-time implementation of a system. The video shows one subject wearing breathing belt on the left side and our GUI on the right side. The experiment setup is shown on the top figure on the GUI. During the experiment, the subject is instructed to change his breathing patterns at specific time instances that will be described next. During the experiment, breathing rate from the reference breathing belt (red line) and estimated breathing rate (blue line) from our radar system were shown on the graph in real-time but with about 18 s delay. In addition, current values of breathing rate, classified breathing pattern (under the label Breathing) and activity status (under the label Status) were shown on the right.

Algorithm 1 Radar selection algorithm

```
function RADAR_SELECTION(sh_l, sh_r, sh_mid, rd_loc)    ▷ Where sh_l - coordinate of left shoulder, sh_r - coordinate of right shoulder, sh_mid - coordinate of middle point of shoulders, rd_loc - three radars' coordinates in the reference system
    for i = 1:3 do
        // check if subject is in each radar's detection area
        if 60° < arctan(|Δz|/|Δx|) ≤ 90° then    ▷ described in Section 4.4 and Figure 4(B)
            rd(i)_ind = 1 //if subject is in this radar's detection area then its index will be 1
        else
            rd(i)_ind = 0 //if subject is not in this radar's detection area then its index will be 0
        end if
    end for
    rd_num = find(rd_ind==1)
    if len(radar_num) = 0 then
        // subject detected by Kinect but not in radar detection area
        Break
    else if len(radar_num) = 1 then
        // subject is in one radar's detection area
        The radar whose index is 1 will be selected for respiratory signal extraction
    else
        // subject is in multiple radars' detection area
        Calculate orientation angle with respect to radars using Equation 3
        The radar whose orientation angle is closest to 90°is selected
    end if
end function

    // Main algorithm
if subject_num = 0 then
    // no subject detected by Kinect then stop the process until a subject detected
    Break
else if subject_num = 1 then
    // one subject detected by Kinect
    Run function RADAR_SELECTION
    Return The selected radar index
else if subject_num = 2 then
    // two subjects detected by Kinect
    for each subject do
        Run function RADAR_SELECTION
    end for
    if radar_sub1 = radar_sub2 then
        // a same radar is selected for both subjects
        Calculate distances between subjects' shoulder middle points and selected radar using Equation 2
        if abs(dist_sub1-dist_sub2) < 0.5m then
            // two subjects are close to each other
            Rerun function Radar_selection
            The radar whose orientation angle is the second closest to 90°is selected for each subject
        else
            Return the selected radar index from function RADAR_SELECTION for each subject
        end if
    else
        Return the selected radar index from function RADAR_SELECTION for each subject
    end if
else
    // more than two subjects detected by Kinect
    Break // This paper focus up to two subjects' respiration monitoring
end if
```

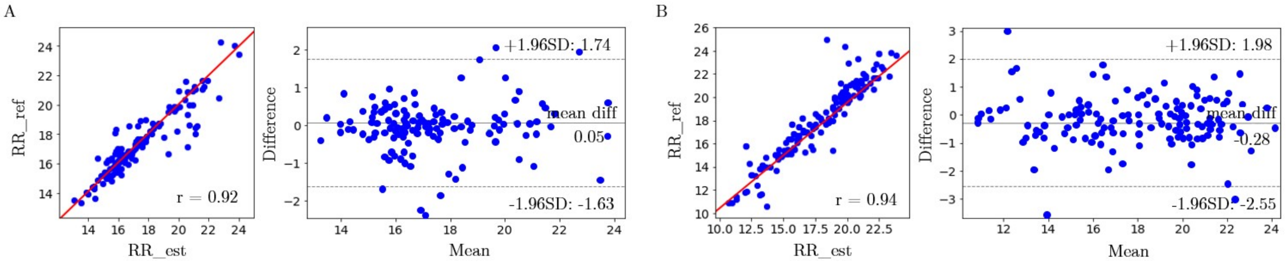

Figure S2. (A) Correlation plot (right) and Bland-Altman plot (left) of the estimated RR and reference RR for one-subject experiments, (B) Correlation plot (right) and Bland-Altman plot (left) of the estimated RR and reference RR for two-subject experiments.

Experiment setup: a male subject was asked to sit at the center of the monitoring zone facing radar 1. He remained stationary during the experiment and performed 4 types of respiratory pattern according to the commands of the experiment coordinator. Next we will describe the activities performed as well as what is observed on the GUI:

- At 0 s (start the recording), the subject breathed normally following the metronome set to 16 beats per minute (bpm). The system showed the corresponding respiratory rate and respiratory pattern (Normal) on the GUI at the start of the recording.
- At 45 s, the experiment coordinator gave a command to perform Cheyne Stokes respiration (CSR). At 50 s, the subject started to perform the CSR.
- At 1 min 20 s, the subject ended the ventilation period of CSR and hold his breath completely to emulate the apnea. The system showed the ”Cheyne Stokes” at 1 min 34 seconds. At 1 min 38 seconds, the system started to displayed apnea on screen. Please note that we focus on estimating breathing rate during normal breathing - therefore when the abnormal breathing pattern detected, the breathing rate is still shown on the graph but it is less accurate due to sudden changes in breathing rate. Also notice that it takes about 40 seconds to detect Cheyne-Stokes breathing patterns because this is the duration of the CSR event. Before the final classification of CSR, the algorithm perform classifications based on 15 s chunks and therefore the system identifies (with 18 s delay) first abnormal breathing and then apnea before it classifies CSR.
- At 1 min 50 seconds, the subject started to breath normally again following the metronome of 16 bpm. At 2 min 14 seconds, the system displayed the corresponding RR and RP on screen.
- At 2 min 45 seconds, the experiment coordinator adjusted the metronome to 29 bpm. The subject increased his breathing frequency and breathing tidal volume and followed the metronome to perform Kussmaul respiration. At 3 min 10 seconds, the system displayed the corresponding RR and RP (Abnormal respiration) on screen.
- At 3 min 27 seconds, the experiment coordinator sets the metronome back to 16 bpm. The subject decreased his breathing frequency and tidal volume, and followed the metronome to breath normally again. At 3 min 54 seconds, the system displayed the corresponding RR and RP on screen. At 4 min 6 seconds, the experiment ended.
